# How Hox genes can shed light on the place of echinoderms among the deuterostomes

**DOI:** 10.1186/2041-9139-5-22

**Published:** 2014-06-17

**Authors:** Bruno David, Rich Mooi

**Affiliations:** 1 UMR CNRS 6282 Biogéosciences, Université de Bourgogne, 21000 Dijon, France; 2 Department of Invertebrate Zoology and Geology, California Academy of Sciences, 94103 San Francisco, California, USA

**Keywords:** Evolution, Development, Hox genes, A/P axis, Deuterostomia, Echinodermata

## Abstract

**Background:**

The Hox gene cluster ranks among the greatest of biological discoveries of the past 30 years. Morphogenetic patterning genes are remarkable for the systems they regulate during major ontogenetic events, and for their expressions of molecular, temporal, and spatial colinearity. Recent descriptions of exceptions to these colinearities are suggesting deep phylogenetic signal that can be used to explore origins of entire deuterostome phyla. Among the most enigmatic of these deuterostomes in terms of unique body patterning are the echinoderms. However, there remains no overall synthesis of the correlation between this signal and the variations observable in the presence/absence and expression patterns of Hox genes.

**Results:**

Recent data from Hox cluster analyses shed light on how the bizarre shift from bilateral larvae to radial adults during echinoderm ontogeny can be accomplished by equally radical modifications within the Hox cluster. In order to explore this more fully, a compilation of observations on the genetic patterns among deuterostomes is integrated with the body patterning trajectories seen across the deuterostome clade.

**Conclusions:**

Synthesis of available data helps to explain morphogenesis along the anterior/posterior axis of echinoderms, delineating the origins and fate of that axis during ontogeny. From this, it is easy to distinguish between ‘seriality’ along echinoderm rays and true A/P axis phenomena such as colinearity within the somatocoels, and the ontogenetic outcomes of the unique translocation and inversion of the anterior Hox class found within the Echinodermata. An up-to-date summary and integration of the disparate lines of research so far produced on the relationship between Hox genes and pattern formation for all deuterostomes allows for development of a phylogeny and scenario for the evolution of deuterostomes in general, and the Echinodermata in particular.

## Background

The cluster of specialized regulatory genes called Hox genes remains one of the most fascinating life science subjects of the last three decades. Bridging the two investigatory realms of developmental and evolutionary biology, the study of Hox genes was a major factor in the emergence of a new field of research, ‘Evo-Devo’ [1]. It is now well known that Hox genes play a fundamental role in the body patterning of bilaterian organisms as diverse as worms, insects, vertebrates, and echinoderms [2-7], and putatively in non-bilaterian animals [8]. A remarkable observation is the degree of similarity in these genes (homologous genes known as orthologues among organisms) across even the most phylogenetically disparate of clades. Additionally remarkable is the fact that Hox genes are organized along chromosomes into one or several clusters (known as paralogues within the same organism). The genes in the clusters are highly conserved, and share closely related sequences among orthologues, attesting to their common ancestry and allowing confident identification of Hox genes, even if this is usually a challenging process in pragmatic terms.

A primary recognition criterion for these genes is the existence of a highly conserved ‘homeobox’ sequence about 180 nucleotides in length expressing a sequence of 60 amino acids (the ‘homeodomain’) that specifically binds to DNA or RNA. In other words, the homeobox codes for this homeodomain in all animals. As it turns out, only some of the genes containing a homeobox are classed as Hox genes. These are genes involved in homeotic mutations, showing that this special subset of the homeobox genes is responsible for establishing major body patterning events in animals. These are now known as Hox genes arranged in the aforementioned cluster.

One of the most conspicuous phenotypic expressions of the Hox cluster is the patterning along the antero-posterior (A/P) axis of bilaterian animals at early stages of their ontogeny. This is another way of saying that Hox cluster expression is a process by which modules are established in developing animals. In the course of development, the modularity corresponds to establishment of organizational subunits. Most metazoans (Ecdysozoa, Lophotrochozoa, Deuterostomia) consist of modules whether they are evident (head - thorax - abdomen in *Drosophila* or *Mus*) or not (for example, in adult tunicates). Among these, the modularity of echinoderms is still a matter of debate and discovery as the most conspicuous modules, such as the radiating arms of a starfish, are not necessarily the most evolutionarily relevant in terms of origins of the phyla in general.

Of course, life has had many millions of years to ‘tinker’ with the Hox gene system, and any cursory examination of the literature will reveal descriptions of several complications. Notable among these is the fact that certain other genes exist that appear to be closely related to the Hox cluster. These are members of a sister complex, the ParaHox cluster [9], or part of the so-called ‘extended Hox cluster’ such as the homeotic, even-skipped *Evx*, and the mesenchyme homeobox *Mox*[10]. Taken together, the various types of genes in the extended cluster have been considered members of a Hox megacluster [11].

**Figure 1:**
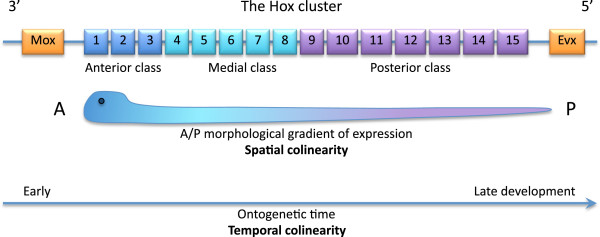
**General organization of a complete Hox cluster with the two additional genes *Mox *and *Evx *(uppermost row).** The Hox genes are grouped into three classes (*Hox3* has been included in the anterior class). Also indicated are expression of the spatial (middle row) and temporal (bottom row) colinearities.

Hox genes themselves are generally arranged into three classes: anterior (*Hox1* and *Hox2*, with *Hox3* arguably falling into its own class); medial (*Hox4* to *Hox8*); and posterior (*Hox9* to *Hox13*, or to *Hox15* in some cases). The anterior-to-posterior sequence of these genes (and therefore also of the anterior, medial, and posterior classes) corresponds to their arrangement along the chromosome, with the anterior class closer to the 3’ end and the posterior closer to the 5’ end of the DNA strand, relatively speaking. The closely related *Evx* flanks the Hox cluster on the 5' side, and *Mox* on its 3' side. This remarkable linearity in organization of the genes along the chomosomes is the basis for a concept known as ‘colinearity’, of which there are two kinds: temporal and spatial. The genes of the anterior class (3' end) are activated first and express earlier in ontogeny than those of the medial class, and the medial are expressed earlier than those of the posterior class at the 5' end, typically in order from *Hox1* to *Hox13*. This is temporal colinearity. In organisms that grow from anterior to posterior, temporal colinearity is in turn transcribed into spatial colinearity, in which the first expressed genes at the 3' end of the cluster code for developmental events in the anterior part of the organism, and the last ones at the 5' end for events in the posterior part (Figure 1).

In combination, temporal and spatial colinearity lead to the expression of a concept called ‘posterior prevalence’, a termed coined by Duboule [12]. That is to say, more posterior genes that are expressed after more anterior ones ‘over-rule’ the medial or anterior class genes in the sense that their body patterning influence is superimposed upon and in fact could be said to dominate the anterior ones. Actually, it is more correct to say that this is a ‘5' end prevalence’ because the last genes to be expressed are towards the 5' end of the chromosome. In a strongly organized cluster, the last expressed genes are generally the most posterior, but some exceptions exist [3].

As suggested earlier, nature is seldom simple, and it is important to note that within this well-organized framework, there are certain exceptions that led [3] to classify Hox clusters into four categories: organized, disorganized, split, and atomized. In basal animals, such as the cnidarian *Clytia*, the cluster seems to be fragmented and displays no colinearity [13]. Other exceptions of importance occur within the superphylum Deuterostomia, in which fully organized clusters are most often found. For example, the appendiculate tunicate *Oikopleura dioica* diplays an atomized cluster. The phlebobranchiate tunicate *Ciona intestinalis* has a split and partly disorganized cluster, which nonetheless does express a residual of colinearity [14,15].

Another major exception concerns the Echinodermata, the primary subject taxon of this paper. The sequencing of the complete genome of the sea urchin *Strongylocentrotus purpuratus*[16], has opened the door to many discoveries dependent on having accurate sequence data. Among these is the realization that the sea urchin Hox cluster is disorganized, and appears to break the rule of spatial colinearity [2]. The principal aim of the present work is to place the Hox cluster of echinoderms in the more general, phylogenetic framework of other deuterostome clades, thereby attempting to establish an evolutionary scenario for the Hox cluster in echinoderms. We propose hypotheses relating the organization of the Hox cluster and some of the associated genes in the extended cluster to the body patterning of echinoderms. How can the disorganization of the cluster be related to anatomic characteristics of echinoderms? What might have been the evolutionary pathways leading to the situation observed in the most crownward echinoderms, the sea urchins? Can major events in echinoderm Hox cluster evolution within the phylum be discussed in a phylogenetic context? To discuss these issues, it will be necessary to establish some unequivocal details through comparisons with other deuterostomes. The present study relies on long-accepted phylogenetic concepts of the Deuterostomia as amplified upon and supported by recent molecular analyses [17,18]. In this interpretation, urochordates appear to be the sister group of craniates with which they have been grouped into the clade Olfactores [19]. Along with the more basal cephalochordates, these constitute the clade Chordata. The sister clade of Chordata, the Ambulacraria, comprises hemichordates plus echinoderms. The enigmatic, possibly primitive, possibly highly reduced, worm-like bilaterian *Xenoturbella* will not be further considered here, as its phylogenetic position as a basal deuterostome remains too controversial [20-22], and has recently been rigorously challenged [23].

The information available regarding Hox genes is difficult to summarize easily because the types of information emphasized in primary sources is disparate and seldom standardized. Most importantly it is vital to take into consideration that the types of study, and therefore the information each transmits, are tightly correlated with content that falls into several main categories that may, or may not be complementary or even congruent: (1) assessment of the presence and characterization of the genes, eventually culminating in a gene tree from which the genes can be identified by empirically discovered membership in a phylogenetically defined group; (2) mapping of genes along chromosomes through sequence analysis; (3) determination of phenotypic expression domains in space and time, usually in early development.

The two first points are relevant to reconstructing the evolution of the Hox cluster. The latter point forms a key to understanding the processes in space and time by which an animal phenotype such as that of an echinoderm is assembled. Science being the contingent process it is, these four categories of data are almost never available to the same degree for each of the five major, extant echinoderm clades (Table 1). Some groups have, for various practical and scientific reasons, been more fully explored than others. Fortunately, the most studied taxa (crinoids, asteroids, echinoids) span most of the phylogenetic divergence of the phylum, and are reasonably good exemplars of the main events in echinoderm evolution at least with respect to the phylogenies presently enjoying the greatest consensus among echinoderm evolutionary biologists. Crinoids are universally considered phylogenetically basal to all other extant echinoderm clades, ophiuroids and asteroids are sister groups, and echinoids and holothuroids are more closely related to each other than to any other group [24]. These observations form the basis of our starting point in organizing information about variations in Hox clusters in the Echinodermata. In order to root the evolution of the cluster, other deuterostomes have been closely examined. As the Ambulacraria has been receiving more and more molecular and even morphological support as a distinct clade, the hemichordates such as *Balanoglossus* constitute the most relevant sister taxa. The sister group to the Ambulacraria is also important to consider, consisting of chordates such the cephalochordate *Branchiostoma*, the urochordates *Ciona* and *Oikopleura*, and the craniates *Danio* or *Mus*.

**Table 1:** Available information regarding Hox genes of major echinoderm clades

	**Crinoids**	**Asteroids**	**Ophiuroids**	**Holothuroids**	**Echinoids**
Gene characterization	PCR [25]	PCR [26-28]	PCR [29,30] Pyrosequencing [33]^a^	PCR [31,32]^a^	Whole genome sequence [2,16]
Mapping of genes along chromosomes	Inferred [30]	Inferred [26]	X	X	Complete translocation - inversion observed [2]
Expression domains in space and time	Embryos Larvae [25,34]	Larvae Juveniles [34-38]	X	X	Embryos Larvae Juveniles [39-43]

The available genetic information is grouped in three main levels (rows) for each echinoderm class (columns). The bottom row gives precision about the developmental stage at which observations were made. Numbers in square brackets correspond to the primary bibliographic references.

^a^Recorded only during regeneration process.

## Results

### Genetic patterns among deuterostomes: outgroups

**Figure 2:**
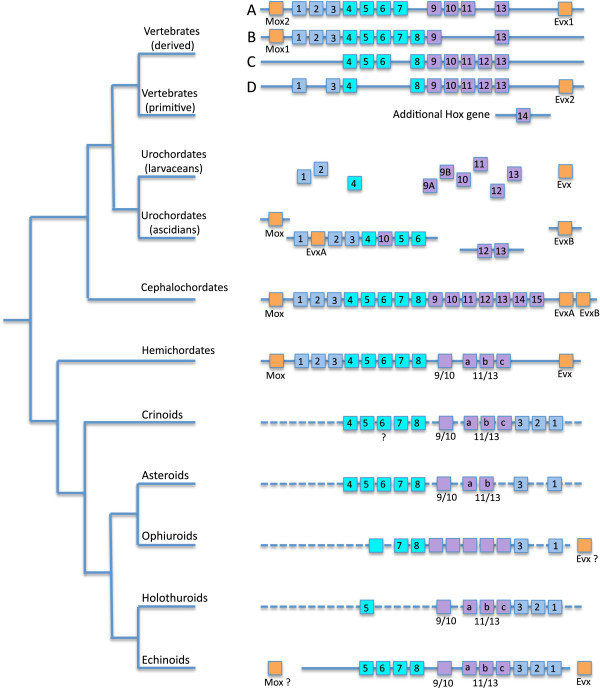
**Arrangement of the Hox cluster in major deuterostome clades.***Evx* and *Mox* genes, located in the vicinity of the Hox cluster, are also indicated. Tree topology is a synthesis of [17,23], and [113] for echinoderm clades. Terminals are based on data from taxa as follows: derived vertebrates on *Mus* (mouse); basal vertebrates on *Lethenteron* (lamprey); larvacean urochordates on *Oikopleura*; ascidian urochordates on *Ciona*; cephalochordates on *Branchiostoma* (amphioxus); hemichordates on *Saccoglossus* (acorn worm); crinoids on a combination of *Metacrinus* (sea lily) and *Oxycomanthus* (feather star); asteroids on a combination of *Asterias* and *Patiriella* (starfish); ophiuroids on *Stegophiura* (brittle star); holothuroids on *Holothuria* (sea cucumber); and echinoids on *Strongylocentrotus* (purple sea urchin). Note that for echinoderms, the alignments for the anterior class are shown in the recently discovered translocated, inverted condition. The nature of the lines through the complex on the right denotes the position of genes on a single (one continuous line), on few (several broken lines), or on many (no line) chromosomes. Putative arrangements are shown by dashed lines.

Even a cursory survey among several non-echinoderm deuterostomes attests to the diversity of their Hox cluster organization, likely adding considerable complexity to their history (Figure 2).

Vertebrates possess multiple, organized clusters with several paralogues for most of the genes (see [44] for a recent review). Chondrichthyans (cartilaginous fishes), sarcopterygians (such as the coelacanth), and mammals (mouse and humans) have four clusters (HoxA to HoxD). In contrast, teleost fishes have several supernumerary clusters, up to eight [45], suggesting that they are apomorphic for this feature within the vertebrates, given that the four clusters found in the elephant shark (*Callorhinchus millii*) are considered representative of the primitive condition for vertebrates [46].

The Hox genes within each paralogous cluster in vertebrates ranges from *Hox1* to *Hox14*. The latter has been identified in the Japanese lamprey and in a catshark [47], as well as in the coelacanth [48] and the lungfish [49], but has not so far been detected in mammals or in teleost fishes. This is suggestive of secondary loss in these lineages. Two paralogues of *Evx* (*Evx1* and *Evx2*) are present at the 5' ends of the HoxA and HoxD vertebrate clusters. Similarly, two paralogues of *Mox, Mox1,* and *Mox2*, exist at the 3' side of *Hoxb1* and *Hoxa1,* respectively [10]. A third paralogue, *Eve1*, exists in teleosteans [50].

Urochordates (ascidians and larvaceans) are the sister group of craniates. Despite this close relationship, their adult gross morphology is strongly divergent from that of their deuterostome relatives as little of the larval bilaterian morphology is retained into adulthood. This highly modified adult morphology seems to be at least partially reflected in the organization of the Hox cluster. *Oikopleura dioica* and *Ciona intestinalis* are the two model species in which Hox clusters have been identified. Each represents one of the two main clades of tunicates [51]. *Oikopleura* has a single copy of the cluster, but it is an atomized (*sensu* Duboule [3]) scattering of nine genes (*Hox1*, *Hox2*, *Hox4*, and *Hox9A, Hox9B* to *Hox13*) distributed throughout the genome. That is, instead of being parts of a relatively cohesive cluster, each Hox gene is surrounded by genes of completely unrelated classes [52]. Only a single type of *Evx* gene has been detected in *Oikopleura. Ciona* is very different [15]. Seven genes (*Hox1* to *Hox6* and *Hox10*) are clustered on the same chromosome, but slightly out of order: *Hox10* is located between *Hox4* and *Hox5*[14]. Two other genes (*Hox12* and *Hox13*) are situated on another chromosome. Therefore, the *Ciona* Hox cluster seems to be partially incomplete, split, and disorganized - but definitely not atomized as in *Oikopleura*. Two *Evx* genes have been identified in *Ciona*, one inserted between *Hox1* and *Hox2*, and the second on another chromosome. In addition, there is a single *Mox* gene that is not located in the vicinity of any Hox [14,53].

Unlike the multiple, paralogous copies found in vertebrates and the disorganization observed in urochordates, the cephalochordate amphioxus (*Branchiostoma*) possesses only a single Hox cluster, and this is of the organized type (*sensu*[3]), with *Hox1* to *Hox14* present [54], along with an additional gene not seen in other deuterostomes, *Hox15*[55,56]. Two *Evx* genes are present at the 5' end of the cluster [55], as well as a single *Mox* gene at the 3' end [10]. *AmphiHox15* seems to be specific to, and an autapomorphy of cephalochordates or perhaps only of *Branchiostoma*.

As one of two clades of ambulacrarians, hemichordates form the reciprocally monophyletic sister group to echinoderms. The acorn worm (*Balanoglossus misakiensis*) possesses a relatively complete Hox cluster arranged in the standard order, with *Hox1* to *Hox8*, *Hox9/10*, *Hox11/13a*, *Hox11/13b*, and *Hox11/13c*[57]. Another species, *Saccoglossus kowalevskii*, has the same set of genes and the same organization, *Hox8*, once regarded as lacking [58], has recently been recognized [59], an observation confirmed by recent study of a third hemichordate species, *Balanoglossus simodensis*, in which *Hox8* has been identified [57]. A similar organization of 12 genes has also been recorded in *Ptychodera flava*[59]. *Hox2*, once reported to be missing in this species [60], has been recently recognized in *Ptychodera*[59] as it is in all other hemichordates (*Balanoglossus* and *Saccoglossus*) studied to date.

These results, taken in context with the basal position of the Ptychoderidae in hemichordate phylogeny [61], support the contention that a full set of 12 Hox genes is ancestral for hemichordates. The Hox-like gene *Mox* has been detected in hemichordates [62], and pending confirmation, *Evx* seems also to be present in *Saccoglossus kowalevskii* (added to GenBank [63], February 2013).

### Genetic patterns among deuterostomes: echinoderms

Echinoderm Hox clusters are very different from those of other deuterostomes, adding considerably to the diversity of an already heterogeneous landscape of Hox configurations (Figure 2).

The echinoid (sea urchin) cluster is by far the most thoroughly investigated in terms of both genetics and within-group diversity, as it has been studied in several species belonging to quite disparate clades. In the camarodont *Strongylocentrotus purpuratus*, the Hox cluster is almost complete, but *Hox4* has not been identified, leaving a total of 11 genes [2]. The peculiarity of the echinoid cluster is not so much the absence of a member in the series, but that the order of the genes themselves along the chromosome is rearranged: *Hox1* to *Hox3* are located at the 5' end of the cluster in reverse order. This topology is the result of a translocation and an inversion so that the resulting pattern is *Hox5* to *Hox9/10* and then *Hox11/13a*, *Hox11/13b*, *Hox11/13c* followed by *Hox3*, *Hox2*, and *Hox1*. As in most other deuterostome configurations, the *Evx* gene flanks the cluster at its 5' end. It is important to note that the aforementioned translocation and inversion places a member of the *Evx* class of gene close to the anterior *Hox1* gene instead of in proximity to a posterior gene. In the irregular echinoid, *Peronella japonica* (Clypeasteroida), the same set of 11 genes was identified, with *Evx* expected at the 5' end of the cluster [64]. It is possible that at least one *Mox* gene also exists in echinoids as a *Mox1*-like homeobox protein has been detected in *S. purpuratus*[63].

Holothuroidea (sea cucumbers) have so far not been considered in as much detail as echinoids regarding their Hox cluster, but a sequence of nine genes has been identified in *Holothuria glaberrima*[31]. It appears that *Hox4* is lacking as well as three other genes of the medial group (*Hox6* to *Hox8*). We note, however, that the authors reported that this part of the cluster might have been underrepresented by the PCR surveys. Moreover, it has been shown that several Hox genes (but not *Hox4*) were involved during the regeneration process of the same sea cucumber species [32], reinforcing the finding that *Hox4* is absent. It has been suggested that *HgHox9* identified by Méndez et al. [31] is actually *Hox9/10*[65], and that *HgHox10* is *Hox11/13a*. In summary, there appears to be a total of eight genes (Figure 2).

Among the asterozoan clade Ophiuroidea (brittlestars), only a preliminary survey is available for *Stegophiura sladeni*[29]. This survey identified one anterior gene (*Hox1*), three medial genes (of which *Hox7* and *Hox8* were positively identified), and putatively five genes of the posterior class. Complementary data from GenBank allow the addition of *Hox3* to the list [30]. A recent exploration of the regeneration process in *Ophionotus victoriae* identified a contig matching an *Even-skipped*-like protein, suggesting the presence of *Evx*[33]. The provisional conclusion is that ophiuroids have a cluster with *Hox1* and *Hox3* in the anterior class, *Hox7* and *Hox8* plus at least one other gene in the medial class, and several genes in the posterior class. The GenBank data were also cited by Fritzsch *et al.*[66] in support of the contention that *Hox4* was absent in ophiuroids. However, these data are in reference to expression of genes during regeneration of *Amphiura filiformis* - this is very different from saying that *Hox4* is absent from the ophiuroid cluster because there is no reason to assume that *Hox4* must reveal its presence during regeneration. Although we do not yet have confirmed data that *Hox4* is present in the Ophiuroidea, there are no unequivocal data to suggest that it is absent, either.

Among the Asteroidea (starfish), several studies on different species permit a robust reconstruction of their Hox cluster. In a very early investigation, seven genes were identified in *Asterias minor*[26]. Another set of seven genes was identified in *Patiriella exigua*, five being orthologues of those in *A. minor*[27]. Still later, it was clearly established that the asteroids *P. exigua* and *A. rubens* both have *Hox4* and *Hox5*[28]. This results in a rather complete sequence of 10 genes: two anterior genes (*Hox1* and *Hox3*, *Hox2* having never been positively identified); five medial genes (*Hox4* to *Hox8*); three posterior genes, two being attributed to *Hox9/10* and *Hox11/13b,* respectively.

Two modern types of Crinoidea (sea lilies and feather stars) have been explored and their Hox cluster is now partially understood. Unfortunately, all the known crinoids studied to date likely belong to only a single clade that internally gave rise to the feather stars [67]. Nevertheless, we are fortunate that one type is a stemmed sea lily, *Metacrinus*, which is relatively basal in comparison to the other, more crownward form, the feather star *Oxycomanthus*. The Hox cluster of the isocrinid, *M. rotundus*, contains eight genes: *Hox1* and *Hox2* for the anterior class, *Hox4*, *Hox5*, *Hox7,* and *Hox8* for the medial class, *Hox9/10* and *Hox11/13c* for the posterior class [25]. *MrHox6* has not been identified. It is likely that the sea lily *Metacrinus* lost the *Hox3* orthologue during diversification after divergence from the feather stars [25].

The comatulid, *O. japonicus*, possesses a cluster of 11 genes. According to the original study by Mito and Endo [29], it departs from the *Metacrinus* cluster in having *Hox3*, one medial gene that seems to correspond to *Hox6* (*Hox4* and *Hox5* having not been identified), and a complete posterior class. As synthesized by Fritzsch *et al.*[66], it is likely that *Oxycomanthus* also has *Hox4*[28], and possibly one of the other medial genes, either *Hox5* or *Hox6*. A conservative, consensus crinoid relying on all the available data would likely possess an almost complete cluster of 11 or 12 genes, depending on future determination of the presence of *Hox6* (Figure 2).

### Body patterning: outgroups

The expression pattern of Hox genes, that is, the Hox code, is one of the most relevant sources of evidence by which to trace in space and time the emergence of major evolutionary novelties. Even if there is some flexibility in the way territories are assigned to specific genes among groups, common patterns still exist, providing clues by which to trace homologues across disparate morphologies. Here we examine some of the Hox body patterning profiles for the major non-echinoderm deuterostome taxa - outgroups that include those on the urochordate-craniate lineage as well as the Echinodermata’s sister group, the hemichordates -- in order to compare these with patterns within the Echinodermata.

#### *Craniates*

In vertebrates, an extensive literature attests to a clear relationship between the organization of the cluster and body patterning (see [68,69] for reviews). The spatial and temporal colinearities are expressed as several gradients that parallel the A/P axis and involve different germ layers. The most conspicuous sites of antero-posterior expression of Hox genes concern: (1) the rhombomeres of the hindbrain and the spinal cord (ectodermic neural tissues), the forebrain and midbrain being specified by other homeobox genes such as *Orthodenticle (Otx)*, *Pax2*, or *Fgf8*[70]; (2) the branchial (pharyngeal) arches (or their derivatives); (3) the somites whose mesodermic portion differentiates into the axial skeleton (vertebrae and ribs); (4) the limb/fin buds, which are difficult to fit into the overall framework because their antero-posterior polarity is not straightforward [71].

In the anterior part of the nervous system, *Otx* expresses in the forebrain and midbrain. Several other genes, including the two paralogues of *Engrailed (En1* and *En2)*, *Fgf8*, *Wnt1,* and genes of the *Pax2/5/8* family, express at the midbrain-hindbrain boundary (MHB). This boundary corresponds to an intervening zone (IZ) between the domains of expression of *Otx* and the *Hox1* paralogues. Going posteriorly, the most 3' paralogue groups of Hox genes (PG1 to PG4) have nested domains of expression in the rhombomeres of the hindbrain [72]. The anterior boundaries of expression of PGs 1-4 map the limits between rhombomeres, more posterior genes expressing along the spinal cord [73]. The anterior limit of the domain of expression of PG5 Hox genes corresponds to the boundary between the hindbrain and the spinal cord [74].

Considering branchial arches, the mandibular arch (BA1) is devoid of any Hox expression, but the most posterior expression of *Otx* reaches this arch [75]. The succeeding arches (BA2 to BA5+) are under the control of PG1 to PG4 Hox genes [76]. At more advanced ontogenetic stages, when supplementary arches differentiate from BA5+, it appears that the anteriormost expression of some PG5 genes starts in BA5 and BA6, and in BA7 for PG6 genes [77].

At the mesodermic level of tetrapod somites, PG3 to PG5 genes control the cervical skeleton, PG5 to PG9 genes the ribcage, Hox genes of PG5 making the neck-ribcage transition. PG10 genes control the lumbosacral region, suppressing the formation of ribs in the most posterior zones of the backbone. This illustrates the ‘posterior’ or 5' end prevalence. PG11 Hox genes regulate the sacral vertebrae by partially suppressing the function of PG10 genes, allowing the formation of the sacral wings [69,71]. PG11 genes are also required for proper patterning of the caudal vertebrae [71,78]. PG13 Hox genes terminate the posterior extension of the body [79]. The PG14 genes, specific to basal vertebrates, show no significant expression in the central nervous system (CNS), but in the hindgut instead [49].

Among craniate clades, the Hox patterning of the hindbrain is shared by animals as different as cyclostomes (lampreys), fishes (teleosts as chondrichthyans), and mammals despite dramatic morphological differences. In all gnathostomes, the expression patterns of Hox genes are also nearly identical along the successive branchial arches however disparate the associated morphologies, such as suspension of the jaw in fishes *versus* middle ear bones in mammals [76]. Beyond their anatomical fate, the somites appear to be under the control of the same PGs. Oulion *et al.*[66] noted that ‘the Hox code in the somitic mesoderm is similar in sharks that have only two sets of vertebrae and in osteichthyan species that have a highly differentiated vertebral column’. This remains true when expanded to tetrapods. The general framework is constant, and second order variations concern the extension of territories (for example, number of vertebrae) and the precise sets of paralogous genes activated for their control (including some redundancy of PGs).

#### *Urochordates*

The amazing Hox gene organizational disparities found in urochordates (see above) makes it difficult to build a coherent, summarizing survey of gene expression in this clade. The most fruitful attempts dealt with the origin of the nervous system in members of two main groups of tunicates, generally *Ciona intestinalis* for ascidians and *Oikopleura dioica* for larvaceans, allowing the delineation of some patterns shared with other deuterostomes [14,52,80]. This was made possible by the anatomy of the neural system of tunicate embryos, which have a distinct ‘head’ region.

Overall CNS formation is controlled by genes whose orthology allows comparisons and identification of homologues among urochordates and with vertebrates. The gene *Otx*, present in vertebrates, has also been identified in urochordates. Ascidians have a single *Otx* copy, while three paralogs (*Otxa*, *Otxb*, and *Otxc)* express in *Oikopleura*[80]. In the presumptive CNS, *Otx* expresses anteriorly, albeit sequentially for its triplicates in larvaceans, and *Hox1* expresses more posteriorly. The respective domains of these genes are separated by an intervening region. The gap separating the territories of these two genes corresponds to the neck region and it can be regarded as equivalent to the IZ in craniates. However, *Pax2/5/8* and *En* are expressed differently in different urochordates. In ascidians, *Pax2/5/8* is always expressed at the level of the IZ [81,82] in a manner similar to that of the *Pax2/5/8* family in vertebrates [83], while *En* is present in the IZ in *Ciona savignyi* but not in *C. intestinalis*. In the latter, it is expressed in two domains on both sides of the IZ [81,84]. In larvaceans (*Oikopleura*), they do not express at all in the IZ, but instead at the anterior tip of the CNS (*Pax2/5/8*), or posteriorly in the caudal ganglion (*En*) [80]. Proceeding posteriorly, in *Ciona intestinalis*, *CiHox1* and *CiHox3* express in the visceral ganglion, and *CiHox5*, *CiHox10,* and *CiHox12* in the tail’s nerve cord, while *CiHox6* expression was not detectable [14]. These genes display a relatively consistent spatial organization, although the temporal colinearity seems to be more disturbed. This might be related to the dispersion of the cluster [14]. In *Oikopleura*, in spite of the atomization of the cluster, the genetic A/P expression along different tissues of the tail (notochord, muscles) follows the order of the paralogues, but the temporal colinearity is no longer respected [52]. It is likely that the breakage of the organization of the cluster along the chromosomes has been facilitated by the loss of a developmental need for temporal colinearity.

In addition to the CNS, a second set of spatial expressions of the Hox genes has been identified in *Ciona intestinalis* involving the gut endoderm of the post-metamorphic juvenile [14]. In this region, *Hox10*, *Hox12,* and *Hox13* express in a coordinated manner.

#### *Cephalochordates*

Expression domain mapping studies are relatively common for the amphioxus, *Branchiostoma*, which is often regarded as archetypical for chordates. Because cephalochordates have a single cluster of Hox genes, their Hox code does not encompass sets of paralogues, and is much simpler than in vertebrates.

In the CNS, cephalochordates share with all urochordates and vertebrates the anterior expression of *Otx* and the posterior expression of *Hox1*. The gene *Otx* controls the cerebral vesicle of the amphioxus, reinforcing its homology with the vertebrate forebrain and, putatively, with the midbrain [74] as well as with the urochordate anterior brain (larvaceans) or brain (ascidians). An intermediate zone, between *Otx* and *Hox1* territories, exists in cephalochordates at the level of the second somite, but without expression of *AmphiPax2/5/8* nor *AmphiEn* genes [74,85]. In fact, *AmphiPax2/5/8* and *AmphiEn* express relatively late, in territories outside the IZ, both posteriorly in the rhombospinal domain at the level of somite 5, and anteriorly at the level of the cerebral vesicle [74,85]. The amphioxus embryonic neural tube lacks evident segmentation, making precise comparisons with vertebrates difficult, except for relative positioning of territories along the A/P axis. Therefore, only a predicted segmentation can be inferred from the regional expression of genes. *AmphiHox1* to *AmphiHox4* are expressed in a very similar, nested pattern in the two amphioxus species [56], close to that observed for their vertebrate orthologues. The most anterior expression of Hox in the nervous system is that of *AmphiHox1* that aligns anteriorly with the middle of the second somite at a level that could be equivalent to the third or fourth vertebrate rhombomere [74,86,87]. Next, *AmphiHox2* is anteriorly aligned with somite 3 (although it has also been observed more posteriorly [88], departing from spatial colinearity), followed by *AmphiHox3* (at mid-somite 4) and *AmphiHox4* (at mid-somite 6). *AmphiHox6* shows variable expression patterns: in *B. floridae* it is posterior to *AmphiHox4*, starting at the limit between somites 6 and 7, as classically expected [87]; in *B. lanceolatum* it expresses more precociously and more anteriorly (half a somite in front of *AmphiHox4)*, hence breaking both colinearity principles [56]. More posterior Hox genes have been investigated only once: *AmphiHox7* and *AmphiHox10* retain temporal colinearity, but they display blurred anterior limits of expression, making it difficult to evaluate their spatial colinearity; *AmphiHox14* expresses in the posterior part of the notochord, but with a rostrally displaced anterior limit, although also diffuse [56].

As a whole, the activation of amphioxus Hox genes respects temporal colinearity starting with *Hox1* at the gastrula stage [56,89]. However, the spatial pattern of expression of the most posterior genes of the cluster have not yet been fully documented. This point deserves future research as this part of the cluster seems highly derived, and ‘even the complete genomic sequence is insufficient to decide whether the posterior Hox genes arose by independent duplications or whether they are true orthologs of the corresponding gnathostome paralog groups’ [90]. Recent comparisons confirm that neither the amphioxus *Hox14*, nor other posterior Hox genes have orthologues in the PGs of vertebrates [49].

#### *Hemichordates*

Many orthologues of chordate homeobox genes, including Hox, have been identified in the direct developing enteropneust balanoglossan, *Saccoglossus kowalevskii*[58]. As discussed for other groups, the nervous system is key, and although hemichordates have a diffuse nervous system, it remains possible to suggest comparisons with the chordate CNS. In chordates, the genes are expressed in relatively restricted regions within the dorsal CNS (see above). Contrastingly, due to the diffuse anatomy of their nervous system, hemichordates express gene territories as bands surrounding the animal that are described as ‘circumferential expression’ [91]. Nevertheless, the expression maps of most genes are similar between chordates and hemichordates despite these dramatic organizational differences. The balanoglossan body consists of three parts in order along the A/P axis: prosome, mesosome, and metasome. In *Saccoglossus*, *Otx* expresses anteriorly in the prosome, and in four more posterior stripes, the latter at the level of the first gill slit on the metasome [91]. This is reminiscent of the posterior limit of expression of *Otx* in chordates at the level of the first branchial arch [75]. There is apparently no trace of the IZ and the territories of *Otx* and *Hox1* abut at the level of the anterior part of the metasome. The *Engrailed* domain slightly overlaps that of *Otx. Pax2/5/8* genes have been identified, but their expression domain is so far almost completely unkown, except for an imprecise signaling of *Pax2* in the anterior part of the metasome [92]. The identified Hox genes express more posteriorly, all in the metasome. *Hox1* is at the level of gill slits, other Hox genes of the anterior and middle classes (*Hox2* to *Hox7*) are a bit more posterior, just anterior to the ciliated band, all almost at the same level depending on the developmental stage considered. *Hox9/10* is at the level of the ciliated band, the three *Hox11/13* paralogues express further backward at the level of the posterior sucker in juveniles [58]. This arrangement fits fairly well the expressions observed in vertebrates, with the *Hox11/13* genes expressing back towards the anus.

### Body patterning: echinoderms

**Figure 3:**
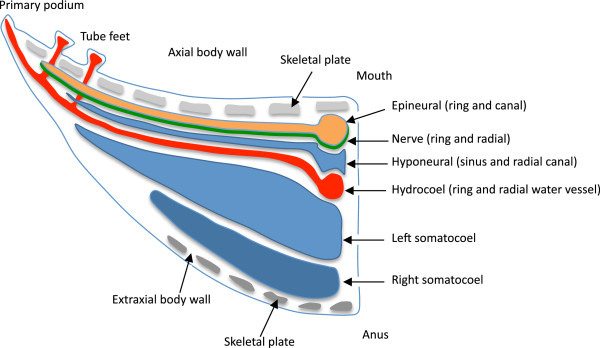
**Diagrammatic cross-section of a young post-metamorphic, generalized echinoderm (only the left side is portrayed).** This presentation synthesizes developmental, morphological, and paleontology data [95,96]. Typically, echinoderms begin development as bilateral embryos or larvae. At metamorphosis, a new structure (the rudiment) develops on the left side of the larva. The elaboration of the rudiment involves three main coelomic cavities, which during metamorphosis turn to stack postero-anteriorly: right somatocoel (dark blue), left somatocoel (light blue), and hydrocoel (red). The hydrocoel takes on a ring shape and produces five radiating extensions (the primary lobes) that will give rise to the primary podia, which will in turn mark the extremity of the five growing radial water vessels. The adult water vascular system, derived from the hydrocoel, comprises the circumoral ring and the radial water vessels. The tube feet (secondary podia) are lateral buds branching on those radial water vessels. In the oral direction, the left somatocoel develops five extensions that proceed through the hydrocoelar ring, separate from the left somatocoel and fuse to form the hyponeural sinus (dental sacs in echinoids). From this sinus will develop the hyponeural radial canals. More anteriorly, the epidermal layer thickens and bends into five epineural folds, which will give rise to the nerves (green) and epineural sinuses (orange), following the same pattern as the hydrocoel: circumoral (epineural and nerve rings), and radial elements (epineural canals and radial nerves). In summary, there are two main regions to a developing echinoderm. One incorporates right and left somatocoels and develops from the non-rudiment region of the larva; it corresponds to the extraxial part of the body (sensu EAT [95,113]). The other incorporates the hydrocoel (water vascular system), the hyponeural elements and the epithelial derivatives (nerves and epineural strands) and is derived from a rudiment that develops laterally on the larva; it corresponds to the axial part of the body (sensu EAT).

‘Examination of the expression pattern may provide a new tool in the quest to explain the evolutionary origins of the echinoderm radial body plan’ [93]. This premonition was likely correct, but the full quest is far from being achieved. In terms of comparing echinoderms with members of other deuterostome phyla, major questions arise quickly because the ‘reorganization of body architecture involved extensive changes in the deployment and role of homeobox genes’ [94]. In addition, only two classes, echinoids and crinoids, have been examined in the ways required to determine gene expression patterns and correlate those with morphogenesis. The studies so far available pose a challenge for developing robust comparisons for two main reasons. One is that echinoids, which have been the most extensively studied, are nevertheless too highly derived among echinoderms to allow uncritical, direct comparisons with deuterostomes outside the Echinodermata [95,96]. The other problem is that the available data obtained from crinoids focus on developmental stages that are too early to be relevant to the problems we are investigating here, as they are not applicable to study of the expression domains of several crucial genes. These limitations suggest that an appropriate way to proceed is to synthesize otherwise valuable findings from disparate studies in order to determine hypothetical ancestral traits for the phylum overall. This is not as unreasonable as it sounds, given that the data available span the spectrum of most basal (crinoids) to most derived (echinoids) of the contained major clades. Furthermore, complementary evidence emerges from single gene investigations in asteroids [35-37]. Figure 3 illustrates some terminology and provides some explanation concerning the developmental events that result in the typical anatomy of echinoderms.

#### Crinoids

**Figure 4:**
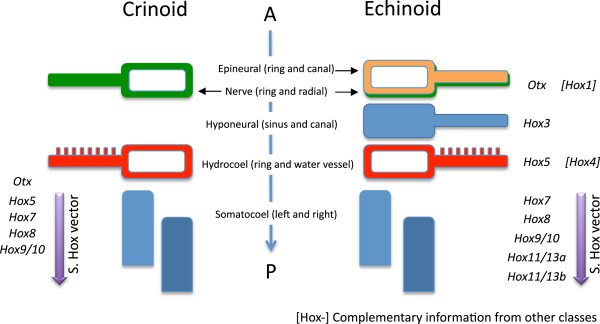
**Comparison of Hox expression domains in crinoids and echinoids.** Figure showing antero-posterior organization of anatomical elements at postmetamorphic stages. Hox gene assignments in square brackets represent complementary data from other taxa. Vertical purple arrows represent the somatocoelar hox vectors.

Among the eight Hox genes identified in pedunculate crinoids (sea lilies), the territories of four have been mapped at early developing stages ranging from hatching embryos to middle auricularia (first larval stage) [25]. The first gene to be expressed is *Hox7* at the end of the gastrula stage, then *Hox8* at the hatching stage, followed by *Hox5* and *Hox9/10* during or slightly before the preauricularia stage. At the preauricularia stage, when all four genes are activated, they are expressed in mesoderm of the incipient somatocoel in an antero-posterior order: *Hox5*, *Hox7*, *Hox8*, and *Hox9/10* (Figure 4). At a later stage (auricularia), the same A/P pattern prevails in parallel in the left and right individualized somatocoels. Earlier, at the hatching stage, *Hox7* expresses along with *Hox8* in the posterior hemisphere of the archenteral sac, which will later develop into the somatocoels. An ectodermal expression of *Hox7* also exists in the oral hood of the auricularia. No trace of expression of the other identified genes (*Hox1*, *Hox2*, *Hox4*, and *Hox11/13c*) has been observed, suggesting that they express still later in development. This is very likely for *Hox11/13c*, which is the most posterior gene of the cluster. A similar situation could also pertain to *Hox1*, *Hox2,* and putatively *Hox4* if we consider that they were translocated to the 5' end of the cluster as is the case in echinoids.

In the same sea lily species, *Metacrinus rotundus*, *Otx* expresses in the archenteron of the gastrula in a position slightly anterior to that of *Hox7* and *Hox8*[34]. At the early auricularia stage, a clear A/P pattern prevails, *Otx* being expressed in the enteric sac, just anterior to the succession *Hox5*, *Hox7*, *Hox8*, and *Hox9/10* in the presumptive somatocoel. Such a pattern of expression of *Otx* in *M. rotundus* is consistent with that observed in other deuterostomes. This is additional evidence evidence that *Hox1* is no longer spatially related to *Otx* as it is in other deuterostomes, supporting the hypothesis of a translocation event in crinoids.

#### Asteroids

Relatively little is known about body patterning of starfish. *Hox1* is expressed in the radial nerve cord and in the circumoral nerve ring of *Asterias rubens*[38]. *Hox4* expression was detected in *Parvulastra exigua*, an asterinid starfish with abbreviated development [36]. The *Hox4* gene expresses successively in the larval coeloms and in the rudiment of the juvenile in the epithelium of the anterior coelom and archenteron of the larva and then in the hydrocoel (including the primary lobes). To a lesser degree, it is also expressed in the right and left somatocoels of the developing rudiment.

In a related species, *Patiriella regularis*, *Otx* has been detected in the fore- and mid-gut regions of the archenteron of the earliest larval stage (bipinnaria) [37]. Slightly later, this gene also expresses in the right and left somatocoels. This early expression in the archenteron of *Patiriella* is comparable with that observed in *Metacrinus*, but without the later expression in the somatocoels. *Engrailed* expresses at different developmental stages in three *Patiriella* species [35]. It has been detected in the developing hydrocoel and in the lining of the somatocoel at a later larval stage (brachiolaria), and in the circumoral nerve ring, and then along the radial nerve in developing juveniles.

#### Echinoids

Ontogenetically, the first Hox activity detected in the sea urchin *Strongylocentrotus purpuratus* was for *Hox7* and *Hox11/13b* at the pregastrulation stage, in the ectoderm and in the foregut [97], but no detailed mapping was provided. Later, in the developing rudiment, the activation of Hox genes starts with *Hox7* followed sequentially by *Hox8* to *Hox11/13b* (Figure 4). The territories of expression of these genes perfectly match an A/P axis along the somatocoels, prompting Arenas-Mena *et al.* to coin the term ‘Hox vector’ to describe this somatocoelar pattern in the mesoderm [39]. This term can be expanded to refer to a more precise term, ‘somatocoelar hox vector’, which makes explicit reference to the somatocoels [96]. Still later, a *Hox3* expression domain maps the hyponeural sinus (dental sacs) in accord with a five-fold pattern [97].

In *Strongylocentrotus droebachiensis*, *Otx* expresses in hydrocoel derivatives, at the level of the circumoral ring and in the primary lobes [94]. *Pax2/5/8* has been identified recently in sea urchin [40]. It expresses at early pre-larval stages in the ectoderm. Nothing is known regarding later developmental stages, and it is not yet certain if it is spatially related to *Otx*.

Additional information comes from observations in direct developing echinoids [41-43,98]. In *Holopneustes purpurascens*, Morris *et al.*[41-43] observed an A/P order of expression of four genes: *HpHox11/13* is the most posterior, its oral termination being aboral (posterior) to the primary podia; *HpHox5* is expressed between tube feet in a relationship with the hydrocoel, anterior to the somatocoels; *HpHox3* is beneath the epithelium of the epineural folds, in the most orally located mesoderm, between the primary lobes of the hydrocoel, in the vicinity of *HpHox5*; *Otx* is the most anterior and it expresses in ectoderm derivatives at the level of the circumoral nerve ring, in the proximal part of the radial nerves, in the epineural fold, and in the ectoderm of the primary lobes of the hydrocoel. Similar observations have been made in *Heliocidaris erythrogramma* and *Phyllocanthus parvispinus*[98]. This is in contrast to indirect developers, in which no pre-metamorphic expression of *Otx* has so far been detected at these sites, suggesting a temporal shift of gene expression between direct and indirect developers.

## Discussion

### The expression of orthodenticle (*Otx*): an anterior marker

In all non-echinoderm deuterostomes, *Otx* expresses anteriorly with regard to all Hox genes. The same general pattern prevails in echinoderms, in which *Otx* seems to express within anterior domains. At early stages it expresses anterior to all Hox genes in crinoids [34], and in the adoral nervous system of echinoids [41]. *Otx* is also closely associated with the hydrocoel. It has been detected in the buds of the primary lobes at pre-metamorphic stages in echinoids, and it is most strongly expressed in the ectoderm of the tube feet in postmetamorphic juveniles of ophiuroids, asteroids, holothuroids, and echinoids [99]. Omori *et al.*’s [34] work on the crinoids concentrates on developmental stages too early to assess the expression of *Otx* in crinoid hydrocoelar elements. Nevertheless, we conclude that *Otx* is a good marker for ‘axial’ elements in the sense of the Extraxial-Axial Theory (EAT) [95]. The involvement of *Otx* in patterning hydrocoelar elements strengthens the postulate that axial (*sensu* the EAT) is relatively anterior, with the extraxial components more posterior, concomitantly delineating an A/P axis [96]. This expression pattern of *Otx* in echinoderms is in complete agreement with observations made for other phyla, such as chordates, arthropods or mollusks, in which *Otx* specifies anterior structures [100,101].

### The intervening zone between *Otx* and *Hox*: an apomorphy for Ambulacraria

**Figure 5:**
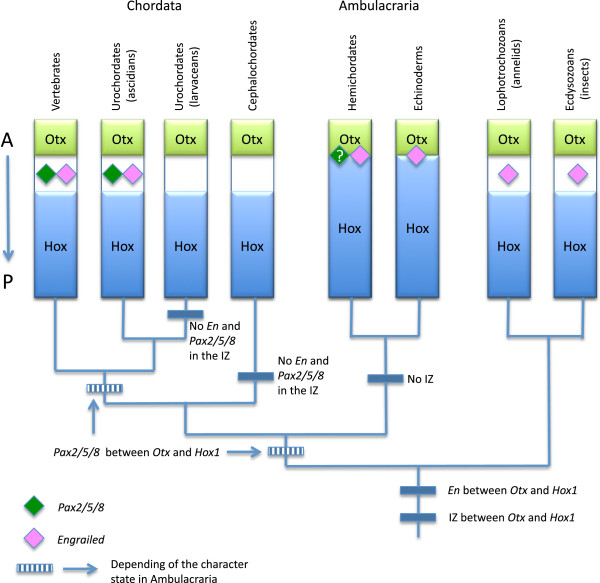
**Expression pattern of the Intervening Zone and of related genes among bilaterian Animalia.** The IZ (separating *Otx* and *Hox1* domains) corresponds to the midbrain-hindbrain boundary (MHB) in vertebrates, the neck region of urochordates, the anterior part of the second somite in cephalochordates, the deutocerebrum of insects, and to the anterior part of the trunk in annelids. Expression domains of *Pax2/5/8* and *En* are shown (diamonds) only when they are located between those of *Otx* and *Hox1*. Expression domains of *Pax2/5/8* are still largely unknown in Ambulacraria, hence the two alternative hypotheses shown for this character.

In some clades, there is a gap between the territory of *Otx* and that of the most anterior Hox, therefore suggesting a tripartite organization, while in other clades the territories abut (Figure 5). When it exists, the intervening zone (IZ) might or might not correspond to the domain of expression of several genes, among them *Pax2/5/8*, *En*, or *Fgf8*.

The craniates have an IZ corresponding to the midbrain-hindbrain boundary region (MHB) in which several genes express. All urochordates share similar general organization with domains of *Otx* and *Hox1* separated by an IZ corresponding to the neck area [102], but ascidians (*Ciona*) and larvaceans (*Oikopleura*) are extremely different in the position and manner in which *Pax2/5/8* and *En* express. Cephalochordates share with all urochordates and vertebrates the anterior expression of *Otx* and the more posterior expression of *Hox1*, with an intervening non-Hox region, but without expression of *Pax2/5/8* nor *En* genes. In hemichordates there is no gap between *Otx* and *Hox1* domains, but *En* expresses close to *Otx* in a position consistent with that of the IZ in the former clades (see above).

In echinoderms, the presence of an intervening zone seems unlikely. Contrary to all chordate clades, but in close similarity to hemichordates, there is no detectable gap between the expression domain of *Otx* and that of the most anterior Hox (*Hox1*, *Hox3*, *Hox5,* or *Hox7* depending of the developmental stage and of the taxa considered). *Engrailed* expression domains, as observed in starfish [35], are relatively anterior, and could be compatible with those of *Otx*. However, there are no studies incorporating these genes in the same set of observations of a single developmental stage and a single class. Therefore, we are restricted to hypothetizing an interaction between *Otx* and *En* domains as it exists in chordates.

In outgroups well outside the deuterostomes, the intervening zone is present in, for example, insects (*Drosophila*). However, the orthologues of *Pax2/5/8* do not express in the IZ, even though they express in the immediate vicinity at the interface between the domains of *Otx* and *Gbx*. In contrast, *En* is present in the neuroblasts of the IZ [103]. Similarly, a gap between *Otx* and *Hox1* seems to exist in annelids (*Platynereis*) with the expression of *Engrailed* at this intermediate level [99]. *Pax2/5/8* expresses in neurogenic domains at very early stages (gastrulation), but in a relatively extended mediolateral band [104]. Later in development, the paralogue *Paxß* expresses along the entire ventral nerve chord in the leech *Helobdella*[105], and therefore outside of and in addition to the IZ.

These observations prompt us to distinguish three main conditions: (1) absence of the IZ in which the domains of *Otx* and *Hox* are contiguous (when identified *Engrailed* domain of expression appears spatially close to that of *Otx*, that of *Pax2/5/8* is still too imprecisely known); (2) presence of an IZ without expression of specific genes (*Pax2/5/8* and *En* orthologues exist, but they express outside this zone); (3) presence of an IZ with expression of specific genes (*Pax2/5/8*, *En* or their orthologues). The first condition seems to apply only to hemichordates and echinoderms, and is likely to be apomorphic for Ambulacraria that can display a derived bipartite organization (Figure 5). Indeed, the existence of a gap is shared by all other deuterostomes (that is, chordates) as well as by ecdysozoan and lophotrochozoan outgroups, and application of the outgroup criterion strongly suggests that this gap is plesiomorphic. The expression of genes in the IZ is more complicated to interpret, as intra-clade variation exists (urochordates). *Engrailed* can be regarded as plesiomorphic as it was identified in non-deuterostome clades, in Ambulacraria as well as in two chordate groups. Therefore, it appears to have been lost independently in cephalochordates and larvacean urochordates. Its expression at the *Otx*-*Hox1* boundary in the hemichordate *Saccoglossus* and in the starfish *Patiriella* suggests that its presence is independent of the existence of an IZ.

### A translocated Hox cluster: an apomorphy for Echinodermata?

It is likely that all echinoderms have a disorganized Hox cluster in a way that allows the most anterior genes to be translocated to the 5' end. Although there is no direct evidence that the disorder exists at earlier nodes (the mapping of the genes is available only for echinoids, see Table 1), it is likely that the cluster exhibits the same pattern of disorder at the base of the clade. This can be logically inferred from the temporal and spatial expression of Hox genes in crinoids, which are the most basal living echinoderms. Indeed, the first gene to be transcribed in pedunculate crinoids is *Hox7*[25], followed by serial expression in the incipient somatocoel involving *Hox5* to *Hox9/10*. None of the most anterior genes (*Hox1* to *Hox4*) is expressed before this serial expression. Taking into account the temporal colinearity rule, this would imply that translocation is also present in crinoids. In addition, in crinoids, the expression domains of *Otx* abut those of *Hox5* or *Hox7* (depending on the developmental stage investigated), while in hemichordates *Otx* and *Hox1* territories are spatially close. Also in other deuterostomes, *Hox1* expresses just posteriorly to the intervening zone. This suggests that, considering the spatial colinearity rule, crinoid *Hox1* to *Hox4* are no longer in the vicinity of *Otx* on the 3' side. These observations strongly support translocation as an apomorphy for all echinoderms.

An ensuing question is: At what point in the cluster is the break point that leads to gene reorganization in echinoderms? If we maintain the assumption that the translocation-inversion is an apomorphy for all echinoderms, then the break is necessarily somewhere between *Hox3* and *Hox5*. With respect to crinoids, it should be anterior to *Hox5*, which is part of the first set of Hox genes involved (member of the ‘somatocoelar Hox vector’). Considering echinoids, it is posterior to *Hox3*, which is itself involved in the translocation. Therefore, two possibilities arise: a break between *Hox3* and *Hox4*, or between *Hox4* and *Hox5*. Both possibilities are consistent with the absence of *Hox4* in echinoids, and correlatively with the suggestion that the translocation preceded this loss of *Hox4*[2]. However, as *Hox4* does not exist in echinoids, the answer hangs on its expression in crinoids that has still to be explored.

### Breaking the rules of colinearity

**Figure 6:**
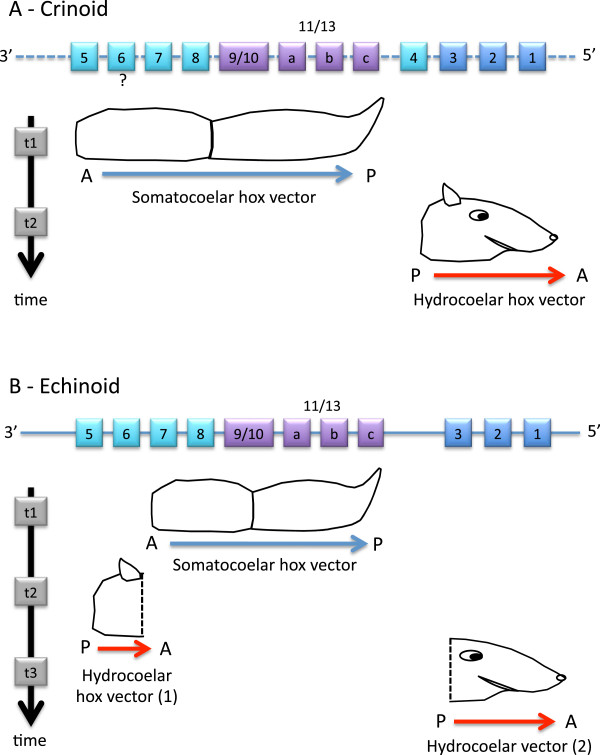
**Departure from the colinearity rules in echinoderms (crinoids and echinoids). (A)** Crinoids. **(B)** Echinoids. The most parsimonious hypothesis regarding the spatial and temporal expression of the Hox genes in crinoids and echinoids indicates that both groups ‘violate’ the spatial colinearity rule as their body (portrayed by a conceptualized bilaterian) is subdivided according to specific Hox expression vectors. The order of Hox genes along the cluster might follow the time vector in crinoids (dashed line indicates that the gene array remains putative), whereas it does not in echinoids in which the temporal colinearity rule is likewise not entirely followed.

The empirical rules of spatial and temporal colinearity are not as universal as formerly suggested, and different animals escape them one way or another. The rules are obviously broken when the Hox cluster is highly disordered or atomized as in the urochordates *Ciona* or *Oikopleura*, but they can also be obviated in more organized clusters. Regarding the cephalochordate *Branchiostoma lanceolatum*, it has been shown recently how a few Hox genes (*Hox6* and *Hox14*) respect neither spatial nor temporal colinearity [56]. In echinoderms, the evolutionary modification of the Hox cluster has also had some consequences for colinearity rules (Figure 6). In crinoids, the temporal colinearity is generally respected, assuming that the anterior genes *Hox1* to *Hox3* have been translocated to the 5' end (see above), but not spatial colinearity. Indeed, the first formed elements are set in A/P order by regulation of the medial and posterior genes that occupy the most 3' positions in the cluster. Then, the anterior genes, translocated to the 5' end and inverted, would be activated later for a reversed (P/A) sequence of the anterior part of the body (Figure 6A). In the more derived echinoids, due to the exclusion of *Hox5* and *Hox6* from the somatocoelar vector (the so-called ‘left behind’ genes *sensu* Mooi and David [96]), the temporal colinearity is likewise no longer respected. The result is a three-interval sequence in which the first genes to be expressed are in the middle of the cluster: (1) expression of *Hox7* to *Hox11/13* in the somatocoelar Hox vector; (2) expression of *Hox5* and possibly *Hox6*; (3) expression of *Hox3* to *Hox1* in another Hox vector, referred to as the ‘hydrocoelar Hox vector’ [96] (Figure 6B).

### Hox cluster territories: the A/P axis is not seriality

All echinoderms follow the same general processes of development. Broad but detailed embryological investigation leads to identification of homologous ‘stacked’ elements (*sensu* Peterson *et al.*[102]) in the five extant classes [95]. Starting opposite the mouth, the most completely expressed pattern (for example, in echinoids) is as follows: the right and left somatocoels, the hydrocoel, the hyponeural sinus, the nerve, the epineural ring (Figure 4). This stacking parallels the A/P axis [39,102]. Meanwhile, many echinoderms display a repetition of elements along the extending radial water vessels. This is particularly visible along the arms in asteroids or ophiuroids as well as along the plate colums in echinoids. We have here specifically restricted the term ‘seriality’ to designate repeated systems along rays that are not directly related to the A/P axis as recently clarified [43].

#### A/P axis

In terms of spatial expression of genes, *Hox1* has been detected at the level of the radial nerve and circumoral nerve ring in asteroids [38]. *Hox3* is expressed at the level of the hyponeural sinus (echinoid dental sacs) [97], and in asteroids *Hox4* has been detected in the hydrocoel [36]. *Hox5* is expressed in close relationship with the hydrocoel in echinoids [41], but also at the anterior tip of the somatocoels in crinoids [25], and to a lesser degree in asteroids. In the somatocoels, the so-called ‘Hox vector’ encompasses *Hox5* to *Hox9/10* in crinoids [25], and *Hox7* to *Hox11/13b* in echinoids [39]. Together, these territories match the stacking of elements described above, particularly if we focus on the supposed anteriormost expression of each gene. Therefore, by comparison with other deuterostomes, it is extremely likely that this array is representative of the A/P axis in echinoderms, as already suggested [96,102]. This is by far the most parsimonious interpretation of the observed patterns of expression and morphogenesis, with the A/P axis of echinoderms orthogonal to the rays, not along them. Concomitantly, it is important to consider the Hox territories of expression along the A/P axis and not serially (along rays). Even if the expression of some genes seems to diminish from an arm tip to its base (for example, *Otx*, *En*) [106], this corresponds to a temporal sequence of expression within an anatomic entity that has to be regarded in the context of its A/P position. In other words, *Otx* and *En* are still expressed as part of a somatic component that is itself situated along an A/P axis. The gradient aspect is a ‘red herring’, as it is superficially mimetic of, but not homologous to what occurs along say, chordate somites.

#### Seriality

Echinoderms are unique in that they produce conspicuously serial elements that are not rostro-caudal as is generally the case in other bilaterians. This highlights the difference between echinoderms and the bilaterality of animals such as *Drosophila* in which seriality is aligned with the A/P axis. Even-skipped (*Evx*) is a good candidate by which to explore this question. In chordates, the Evx genes display two interesting characteristics: they contribute to dorso-ventral patterning and they are associated with segmentation of the tail region. In cephalochordates (amphioxus), *AmphiEvxA* is involved in dorso-ventral patterning [107]. The same situation pertains to vertebrates [50,108]. In the zebrafish, the paralogue *Evx1* is chronologically transcribed in increasingly ventral interneuronal subsets, thereby moving apart on both sides of the A/P axis, from the dorsal part of the spinal cord [109]. Due to their position at the 5' end of the Hox cluster, Evx genes also have a role in patterning the extreme posterior regions of bilaterian embryos in a way similar to that of Hox genes [110].

The recorded dual capacity of Evx genes to promote segmentation and to be expressed orthogonally to the A/P axis in chordates, even if both expression patterns do not occur at the same place, leads to speculation about whether their orthologue in echinoderms could play a similar role. The seriality observed along echinoderm rays associates repetition of elements with nearly orthogonal deviation from the A/P axis. Therefore, *Evx* could be involved in the formation of rays. However, those hypotheses remain to be tested. Asterozoans would be the right group upon which to perform such tests because the rays start to form early enough to accurately trace the gene territories during their development.

## Conclusion

### Scenarios for evolution of the Hox cluster in deuterostomes

**Figure 7:**
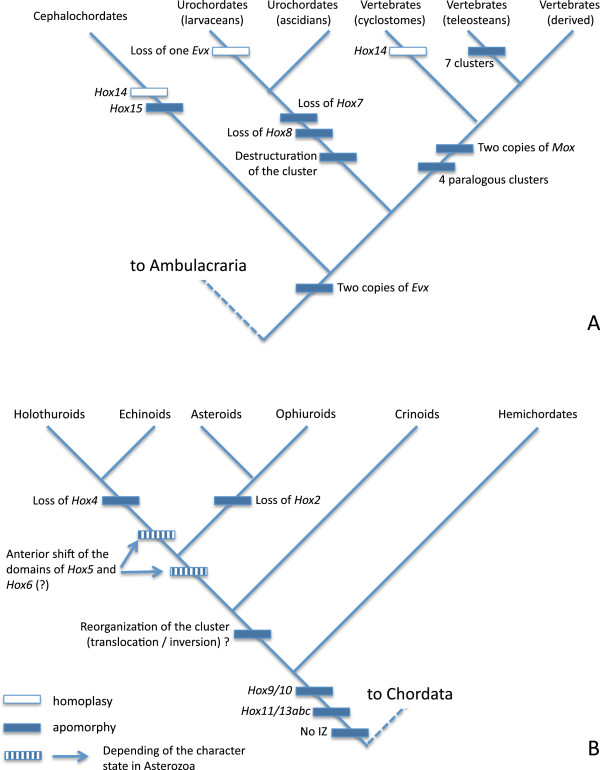
**Phylogenetic mapping of important changes in the evolution of the deuterostome Hox cluster. (A)** Chordata. **(B)** Ambulacraria.*Hox1* has been identified in all deuterostomes. *Hox2* is present in all but asteroids and ophiuroids, suggesting that its absence, if confirmed, is a synapomorphy for the asterozoan clade. *Hox3* has not been identified in pedunculate crinoids, nor in the larvacean *Oikopleura. Hox4* is present in all the groups considered herein, except in the crownward clade grouping echinoids and holothuroids. *Hox5* is missing in *Oikopleura*, possibly in comatulid crinoids, and putatively in the poorly known ophiuroids. *Hox6* has not been detected in *Oikopleura*, pedunculate crinoids, and it is possibly also absent in comatulids, ophiuroids, and holothuroids. The domains of expression of *Hox5* and perhaps *Hox6* could have been shifted anteriorly in some echinoderms; the position of this apomorphy on the tree depends of the character state in Asterozoa. *Hox7* and *Hox8* are present in all deuterostomes except two groups of urochordates and perhaps holothuroids. Their absence in two urochordate clades suggests that loss of *Hox7* and *Hox8* is autapomorphic for this phylum. *Hox9* and *Hox10* have been identified in all chordates with the sole exception that *Hox9* is missing in the urochordate *Ciona*[114]. In Ambulacraria, all groups have a *Hox9/10* gene. *Hox11, Hox12*, and *Hox13* are present in all chordates with the exception of *Ciona*, which is lacking *Hox11*[114], but Ambulacraria have *Hox 11/13abc* genes not ortholog with those of chordates [56]. *Hox14* is convergent between cephalochordates and cyclostomes. *Hox15* is exclusive to cephalochordates. Among the related genes of the extended Hox cluster, *Mox* could be present in all deuterostome clades, pending further explorations in echinoderms, with two paralogous copies found only in vertebrates. *Evx* is present in all metazoans, including clades as basal as cnidarians [13], but two copies exist in all chordate groups (presumed apomorphy), except in larvaceans (presumed reversion).

The available data are robust enough to be mapped, at least in a preliminary manner, on a consensus phylogeny of deuterostomes (Figure 7). This allows us to draw several important conclusions regarding the evolution of the echinoderm Hox cluster. (1) The common ancestor of echinoderms and hemichordates likely had a full set of 12 hox genes [57,59], arranged in a single, organized cluster. (2) The absence of *Hox4* is only confirmed for echinoids and holothuroids. This, added to the robustly determined presence of *Hox4* in crinoids and asteroids strongly suggests that the ancestral echinoderm cluster had *Hox4*, and that the loss of *Hox4* occured after the divergence of echinozoans from asterozoans [59]. The loss of this gene can be regarded as a tentative synapomorphy of the echinozoans only, thus answering the question raised by Pascual-Anaya *et al.*[44] concerning the level of universality of this loss. (3) It has been suggested that in echinoids, ‘the breakage of the cluster is responsible for the loss of *Hox4’*[2]. Owing to new available data, it is likely that the translocation event (breakage) predates the loss of *Hox4*, as the translocation is likely to exist in all echinoderms (see above), and as this loss pertains only to the crownward echinozoans. This observation strongly supports the hypothesis of a translocation-inversion involving at least *Hox1* to *Hox4*, an event that occurred earlier than the loss of *Hox4* in echinozoans (Figure 7). (4) The expression pattern of *Hox5*, and putatively *Hox6*, differs between crinoids and echinoids (Figure 6). In the crinoid *Metacrinus rotundus*, which represents the plesiomorphic condition, *MrHox5* is part of the somatocoelar Hox vector. In echinoids the available data suggest a more anterior condition, *HpHox5* being expressed in a pentaradial pattern between the nascent tube feet [41]. The most parsimonious scenario is that the territories of expression of the echinoid genes *Hox5* (and possibly *Hox6*) have been shifted anteriorly. This event must have taken place in the phylogeny between the crinoid and echinoid nodes. (5) Hemichordates possess three *Hox11/13* genes, and a complete set of these three genes is documented for comatulid crinoids, holothuroids, and echinoids [66]. Despite the fact that some echinoderms (stalked crinoids and asteroids) seem to lack subsets of these genes, Peterson suggested that their presence could represent an apomorphy for Ambulacraria [60]. This hypothesis has been strengthened by Freeman *et al.* who assessed the strong relationship between hemichordate and echinoderm *Hox11/13a,b,c* as well as the lack of orthology with the vertebrate posterior class [59]. More generally, the genes of the posterior class cannot be regarded as homologous among chordates [56], nor can they be homologous between any chordate and the Ambulacraria.

The evolution of echinoderms since the Cambrian is broadly characterized by an increasing expression of axial elements inherited from the rudiment at the expense of extraxial elements derived from the larval body [111,112]. Phylogenetically, the suggested story of the Hox cluster fits with a more pronounced expression of the hydrocoelar Hox vector, and correlatively of axial elements. Owing to the fact that the module derived from the rudiment (axial) represents a more significant portion of the body in echinoids than in crinoids, it makes sense of the fact that there are more genes involved in this module, as suggested by the shift of the territories of expression of *Hox5* and possibly, of *Hox6*.

A plausible evolutionary scenario would place the reorganization of the cluster (translocation-inversion breaking the spatial colinearity rule) at the origin of the clade during or even slightly before the early Cambrian. A supplementary disorganization ensued, with a shift in the expression of *Hox5* and *Hox6*. Pending future examination of the expression domains of these genes in asterozoans, two scenarios are equally probable. The expression shift is related to the loss of *Hox4*, suggesting that it would be apomorphic for the holothuroid-echinoid clade. Another hypothesis would be to relate this Hox cluster event - breaking the temporal colinearity rule - with the loss of the imperforate extraxial body wall at the base of the asteroid-ophiuroid-echinoid-holothuroid clade during the Ordovician.

### Why do echinoderms look so bizarre? A hypothesis to be tested

The most anterior genes are likely related to elements associated with, or anterior to, the hydrocoelar compartment. The translocation-inversion that shifts those most anterior genes to the 5' end of the cluster also results in their prevalence. This means that the expression pattern of the anterior-most compartment of the body would dominate. Because the axial module (sensu EAT) is this anterior-most compartment, and because it is anatomically built according to a radiating five-fold symmetry originating in the five primary lobes of the hydrocoel [95,96], the prevalence of the anterior genes could be related to a dominant pentaradial phenotypic expression. The prevalence shift, related to the activation of rostral genes after the caudal ones, would have changed the balance of expression in body parts, making the five-fold symmetry more dominant without indicating the origins of this radial symmetry.

As already emphasized by Duboule [3], departures from the rule make sense when faced with lineage-specific body patterning. With respect to echinoderms, ‘the disorganization of the cluster might not reflect a release of some constraints […], but might have been a necessary step to escape the obligation of activating “caudal” genes after “rostral” genes, thus favoring, or accompanying, the shift to another developmental mode’ [3]. In this sense, the reorganization of the echinoderm Hox cluster could be reflective of the originality of the echinoderm body plan relative to that of other bilateralians.

## Abbreviations

A/P: Anterio-posterior; BA: Branchial arch; CNS: Central nervous system; EAT: Extraxial-axial theory; IZ: Intervening zone; MHB: Midbrain-hindbrain boundary; PG: Paralogue group.

## Competing interests

The authors (BD and RM) declare that they have no competing interests.

## Authors’ contributions

Both authors (BD and RM) contributed equally to the scientific content and production of this paper. Both authors read and approved the final manuscript.

## References

[B1] Carrol SB Endless forms most beautiful. The new science of evo devo and the making of the animal kingdom 2005 London, New-York: W.W. Norton & Company

[B2] Cameron RA Rowen L Nesbitt R Bloom S Rast JP Berney K Arenas-Mena C Martinez P Lucas S Richardson PM Davidson EH Peterson KJ Hood L Unusual gene order and organization of the sea urchin Hox cluster J Exp Zool B Mol Dev Evol 2006 306B 45 58 10.1002/jez.b.21070 16116652

[B3] Duboule D The rise and fall of Hox gene clusters Development 2007 134 2549 2560 10.1242/dev.001065 17553908

[B4] Kulakova M Bakalenko N Novikova E Cook CE Eliseeva E Steinmetz PRH Kostyuchenko RP Dondua A Arendt D Akam M Andreeva T Hox gene expression in larval development of the polychaetes *Nereis virens* and *Platynereis dumerilii* (Annelida, Lophotrochozoa) Dev Genes Evol 2007 217 39 54 10.1007/s00427-006-0119-y 17180685

[B5] Gehring WJ Kloter U Suga H Evolution of the Hox gene complex from an evolutionary ground state Curr Top Dev Biol 2009 88 35 61 19651301 10.1016/S0070-2153(09)88002-2

[B6] Ikuta T Evolution of invertebrate deuterostomes and Hox/ParaHox genes Genomics Proteomics Bioinformatics 2011 9 77 96 10.1016/S1672-0229(11)60011-9 21802045 PMC5054439

[B7] Bakalenko NI Novikova EL Nesterenko AY Kulakova MA Hox gene expression during postlarval development of the polychaete *Alitta (Nereis) virens* Evodevo 2013 4 13 10.1186/2041-9139-4-13 23635090 PMC3734159

[B8] Ryan JF Mazza ME Pang K Matus DQ Baxevanis AD Martindale MQ Finnerty JR Pre-bilaterian origins of the Hox cluster and the Hox code: evidence from the sea anemone Nematostella vectensis. PLoS One 2007 2 e153 10.1371/journal.pone.0000153 PMC1779807 17252055

[B9] Brooke NM Garcia-Fernandez J Holland PWH The ParaHox gene cluster is an evolutionary sister of the Hox gene cluster Nature 1998 392 920 922 10.1038/31933 9582071

[B10] Minguillon C Garcia-Fernandez J Genesis and evolution of the Evx and Mox genes and the extended Hox and ParaHox gene clusters Genome Biol 2003 4 R12 10.1186/gb-2003-4-2-r12 12620122 PMC151302

[B11] Garcia-Fernandez J The genesis and evolution of homeobox gene clusters Nat Rev Genet 2005 6 881 892 16341069 10.1038/nrg1723

[B12] Duboule D Patterning in the vertebrate limb Curr Opin Genet Dev 1991 1 211 216 10.1016/S0959-437X(05)80072-3 1688004

[B13] Chiori R Jager M Denker E Wincker P Da Silva C Le Guyader H Manuel M Queinnec E Are Hox genes ancestrally involved in axial patterning? Evidence from the hydrozoan *Clytia hemisphaerica* (Cnidaria) PLoS One 2009 4 e4231 10.1371/journal.pone.0004231 19156208 PMC2626245

[B14] Ikuta T Yoshida N Satoh N Saiga H *Ciona intestinalis* Hox gene cluster: its dispersed structure and residual colinear expression in development Proc Natl Acad Sci U S A 2004 101 15118 15123 10.1073/pnas.0401389101 15469921 PMC524048

[B15] Caputi L Borra M Andreakis N Biffali E Sordino P SNPs and Hox gene mapping in *Ciona intestinalis* BMC Genomics 2008 9 39 10.1186/1471-2164-9-39 18221512 PMC2262895

[B16] Sea Urchin Genome Sequencing Consortium The genome of the sea urchin *Strongylocentrotus purpuratus* Science 2006 314 941 952 17095691 10.1126/science.1133609 PMC3159423

[B17] Hejnol A Obst M Stamatakis A Ott M Rouse GW Edgecombe GD Martinez P Baguna J Bailly X Jondelius U Wiens M Muller WE Seaver E Wheeler WC Martindale MQ Giribet G Dunn CW Assessing the root of bilaterian animals with scalable phylogenomic methods P R Soc B 2009 276 4261 4270 10.1098/rspb.2009.0896 PMC2817096 19759036

[B18] Mallatt J Craig CW Yoder MJ Nearly complete rRNA genes from 371 Animalia: updated structure-based alignment and detailed phylogenetic analysis Mol Phylogenet Evol 2012 64 603 617 10.1016/j.ympev.2012.05.016 22641172

[B19] Delsuc F Brinkmann H Chourrout D Philippe H Tunicates and not cephalochordates are the closest living relatives of vertebrates Nature 2006 439 965 968 10.1038/nature04336 16495997

[B20] Lowe CJ Pani AM Animal evolution: a soap opera of unremarkable worms Curr Biol 2011 21 R151 R153 10.1016/j.cub.2010.12.017 21334293

[B21] Philippe H Brinkmann H Copley RR Moroz LL Nakano H Poustka AJ Wallberg A Peterson KJ Telford MJ Acoelomorph flatworms are deuterostomes related to *Xenoturbella* Nature 2011 470 255 10.1038/nature09676 21307940 PMC4025995

[B22] Rottinger E Lowe CJ Evolutionary crossroads in developmental biology: hemichordates Development 2012 139 2463 2475 10.1242/dev.066712 22736243

[B23] Edgecombe GD Giribet G Dunn CW Hejnol A Kristensen RM Neves RC Rouse GW Worsaae K Sorensen MV Higher-level metazoan relationships: recent progress and remaining questions Org Divers Evol 2011 11 151 172 10.1007/s13127-011-0044-4

[B24] Tree of life web project [http://www.tolweb.org/tree/]

[B25] Hara Y Yamaguchi M Akasaka K Nakano H Nonaka M Amemiya S Expression patterns of Hox genes in larvae of the sea lily *Metacrinus rotundus* Dev Genes Evol 2006 216 797 809 10.1007/s00427-006-0108-1 17013610

[B26] Mito T Endo K A PCR survey of hox genes in the sea star: Asterina minor Mol Phylogenet Evol 1997 8 218 224 10.1006/mpev.1997.0417 9299226

[B27] Long SC Morris VB Byrne M Seven Hox gene sequences from the asterinid starfish *Patiriella exigua* (Echinodermata: Asteroidea) Hydrobiologia 2000 420 95 98 10.1023/A:1003937704788

[B28] Long S Martinez P Chen WC Thorndyke M Byrne M Evolution of echinoderms may not have required modification of the ancestral deuterostome HOX gene cluster: first report of *PG4* and *PG5* Hox orthologues in echinoderms Dev Genes Evol 2003 213 573 576 10.1007/s00427-003-0355-3 13680225

[B29] Mito T Endo K PCR survey of Hox genes in the crinoid and ophiuroid: evidence for anterior conservation and posterior expansion in the echinoderm hox gene cluster Mol Phylogenet Evol 2000 14 375 388 10.1006/mpev.1999.0707 10712843

[B30] Martinez P Rast JP Arenas-Mena C Davidson EH Organization of an echinoderm Hox gene cluster Proc Natl Acad Sci U S A 1999 96 1469 1474 10.1073/pnas.96.4.1469 9990047 PMC15486

[B31] Mendez AT Roig-Lopez JL Santiago P Santiago C Garcia-Arraras JE Identification of Hox gene sequences in the sea cucumber *Holothuria glaberrima* Selenka (Holothuroidea: Echinodermata) Mar Biotechnol 2000 2 231 240 10.1007/s101269900027 10852801

[B32] Ortiz-Pineda PA Ramirez-Gomez F Perez-Ortiz J Gonzalez-Diaz S Santiago-De Jesus F Hernandez-Pasos J Del Valle-Avila C Rojas-Cartagena C Suarez-Castillo EC Tossas K Mendez-Merced AT Roig-Lopez JL Ortiz-Zuazaga H Garcia-Arraras JE Gene expression profiling of intestinal regeneration in the sea cucumber BMC Genomics 2009 10 262 10.1186/1471-2164-10-262 19505337 PMC2711116

[B33] Burns G Thorndyke MC Peck LS Clark MS Transcriptome pyrosequencing of the Antarctic brittle star *Ophionotus victoriae* Mar Genom 2013 9 9 15 10.1016/j.margen.2012.05.003 23904059

[B34] Omori A Akasaka K Kurokawa D Amemiya S Gene expression analysis of *Six3*, *Pax6*, and *Otx* in the early development of the stalked crinoid *Metacrinus rotundus* Gene Expr Patterns 2011 11 48 56 10.1016/j.gep.2010.09.002 20837165

[B35] Byrne M Cisternas P Elia L Relf B *Engrailed* is expressed in larval development and in the radial nervous system of *Patiriella* sea stars Dev Genes Evol 2005 215 608 617 10.1007/s00427-005-0018-7 16163500

[B36] Cisternas P Byrne M Expression of *Hox4* during development of the pentamerous juvenile sea star: Parvulastra exigua Dev Genes Evol 2009 219 613 618 10.1007/s00427-010-0318-4 20182887

[B37] Elia L Cisternas P Byrne M Characterization and expression of a sea star *otx* ortholog (*Protxß1/2*) in the larva of *Patiriella regularis* Gene Expr Patterns 2010 10 323 327 10.1016/j.gep.2010.07.003 20647060

[B38] Thorndyke MC Chen WC Beesley PW Patruno M Molecular approach to echinoderm regeneration Microsc Res Techniq 2001 55 474 485 10.1002/jemt.1192 11782076

[B39] Arenas-Mena C Cameron AR Davidson EH Spatial expression of Hox cluster genes in the ontogeny of a sea urchin Development 2000 127 4631 4643 11023866 10.1242/dev.127.21.4631

[B40] McIntyre DC Seay NW Croce JC McClay DR Short range Wnt5 signaling initiates specification of sea urchin posterior ectoderm Development 2013 140 4881 4889 10.1242/dev.095844 24227654 PMC3848187

[B41] Morris VB Byrne M Involvement of two Hox genes and *otx* in echinoderm body-plan morphogenesis in the sea urchin *Holopneustes purpurescens* J Exp Zool B Mol Dev Evol 2005 304B 456 467 10.1002/jez.b.21065 16075458

[B42] Morris VB Zhao JT Shearman DCA Byrne M Frommer M Expression of an *Otx* gene in the adult rudiment and the developing central nervous system in the vestibula larva of the sea urchin *Holopneustes purpurescens* Int J Dev Biol 2004 48 17 22 10.1387/ijdb.15005570 15005570

[B43] Morris VB Byrne M Oral–aboral identity displayed in the expression of *HpHox3* and *HpHox11 /13* in the adult rudiment of the sea urchin *Holopneustes purpurescens* Dev Genes Evol 2014 224 1 11 10.1007/s00427-013-0457-5 24129745

[B44] Pascual-Anaya J D’Aniello S Kuratani S Garcia-Fernandez J Evolution of Hox gene clusters in deuterostomes BMC Dev Biol 2013 13 26 10.1186/1471-213X-13-26 23819519 PMC3707753

[B45] Crow KD Stadler PF Lynch VJ Amemiya C Wagner GP The "fish-specific" Hox cluster duplication is coincident with the origin of teleosts Mol Biol Evol 2006 23 121 136 16162861 10.1093/molbev/msj020

[B46] Ravi V Lam K Tay BH Tay A Brenner S Venkatesh B Elephant shark (*Callorhinchus milii*) provides insights into the evolution of Hox gene clusters in gnathostomes Proc Natl Acad Sci U S A 2009 106 16327 16332 10.1073/pnas.0907914106 19805301 PMC2752591

[B47] Kuraku S Takio Y Tamura K Aono H Meyer A Kuratani S Noncanonical role of *Hox14* revealed by its expression patterns in lamprey and shark Proc Natl Acad Sci U S A 2008 105 6679 6683 10.1073/pnas.0710947105 18448683 PMC2373320

[B48] Amemiya CT Powers TP Prohaska SJ Grimwood J Schmutz J Dickson M Miyake T Schoenborn MA Myers RM Ruddle FH Stadler PF Complete HOX cluster characterization of the coelacanth provides further evidence for slow evolution of its genome Proc Natl Acad Sci U S A 2010 107 3622 3627 10.1073/pnas.0914312107 20139301 PMC2840454

[B49] Feiner N Ericsson R Meyer A Kuraku S Revisiting the origin of the vertebrate *Hox14* by including its relict sarcopterygian members J Exp Zool B Mol Dev Evol 2011 316B 515 525 10.1002/jez.b.21426 21815265

[B50] Cruz C Maegawa S Weinberg ES Wilson SW Dawid IB Kudoh T Induction and patterning of trunk and tail neural ectoderm by the homeobox gene *eve1* in zebrafish embryos Proc Natl Acad Sci U S A 2010 107 3564 3569 10.1073/pnas.1000389107 20142486 PMC2840505

[B51] Tsagkogeorga G Turon X Hopcroft RR Tilak MK Feldstein T Shenkar N Loya Y Huchon D Douzery EJP Delsuc F An updated 18S rRNA phylogeny of tunicates based on mixture and secondary structure models BMC Evol Biol 2009 9 187 10.1186/1471-2148-9-187 19656395 PMC2739199

[B52] Seo HC Edvardsen RB Maeland AD Bjordal M Jensen MF Hansen A Flaat M Weissenbach J Lehrach H Wincker P Reinhardt R Chourrout D Hox cluster disintegration with persistent anteroposterior order of expression in *Oikopleura dioica* Nature 2004 431 67 71 10.1038/nature02709 15343333

[B53] Wada S Tokuoka M Shoguchi E Kobayashi K Di Gregorio A Spagnuolo A Branno M Kohara Y Rokhsar D Levine M Saiga H Satoh N Satou Y A genomewide survey of developmentally relevant genes in *Ciona intestinalis* - II: Genes for homeobox transcription factors Dev Genes Evol 2003 213 222 234 10.1007/s00427-003-0321-0 12736825

[B54] Minguillon C Gardenyes J Serra E Castro LF Hill-Force A Holland PW Amemiya CT Garcia-Fernandez J No more than 14: the end of the amphioxus Hox cluster Int J Biol Sci 2005 1 19 23 15951846 10.7150/ijbs.1.19 PMC1140354

[B55] Holland LZ Albalat R Azumi K Benito-Gutierrez E Blow MJ Bronner-Fraser M Brunet F Butts T Candiani S Dishaw LJ Ferrier DE Garcia-Fernandez J Gibson-Brown JJ Gissi C Godzik A Hallbook F Hirose D Hosomichi K Ikuta T Inoko H Kasahara M Kasamatsu J Kawashima T Kimura A Kobayashi M Kozmik Z Kubokawa K Laudet V Litman GW McHardy AC The amphioxus genome illuminates vertebrate origins and cephalochordate biology Genome Res 2008 18 1100 1111 10.1101/gr.073676.107 18562680 PMC2493399

[B56] Pascual-Anaya J Adachi N Alvarez S Kuratani S D’Aniello S Garcia-Fernandez J Broken colinearity of the amphioxus Hox cluster Evodevo 2012 3 28 10.1186/2041-9139-3-28 23198682 PMC3534614

[B57] Urata M Tsuchimoto J Yasui K Yamaguchi M The *Hox8* of the hemichordate *Balanoglossus misakiensis* Dev Genes Evol 2009 219 377 382 10.1007/s00427-009-0297-5 19657669

[B58] Aronowicz J Lowe CJ Hox gene expression in the hemichordate *Saccoglossus kowalevskii* and the evolution of deuterostome nervous systems Integr Comp Biol 2006 46 890 901 10.1093/icb/icl045 21672793

[B59] Freeman R Ikuta T Wu M Koyanagi R Kawashima T Tagawa K Humphreys T Fang GC Fujiyama A Saiga H Lowe C Worley K Jenkins J Schmutz J Kirschner M Rokhsar D Satoh N Gerhart J Identical genomic organization of two hemichordate Hox clusters Curr Biol 2012 22 2053 2058 10.1016/j.cub.2012.08.052 23063438 PMC4524734

[B60] Peterson KJ Isolation of Hox and Parahox genes in the hemichordate *Ptychodera flava* and the evolution of deuterostome Hox genes Mol Phylogenet Evol 2004 31 1208 1215 10.1016/j.ympev.2003.10.007 15120410

[B61] Cannon JT Rychel AL Swalla BJ Halanych KM Hemichordate evolution: Derived body plans and suspect families Integr Comp Biol 2009 49 E27 E27

[B62] Lowe CJ Terasaki M Wu M Freeman RM Runft L Kwan K Haigo S Aronowicz J Lander E Gruber C Smith M Kirschner M Gerhart J Dorsoventral patterning in hemichordates: Insights into early chordate evolution PLoS Biol 2006 4 9 1603 1619 10.1371/journal.pbio.0040291 PMC1551926 16933975

[B63] GenBank [http://www.ncbi.nlm.nih.gov/genbank/]

[B64] Hano Y Hayashi A Yamaguchi S Yamaguchi M Hox genes of the direct-type developing sea urchin *Peronella japonica* Zool Sci 2001 18 353 359 10.2108/zsj.18.353 11191326

[B65] Long S Byrne M Evolution of the echinoderm Hox gene cluster Evol Dev 2001 3 302 311 10.1046/j.1525-142X.2001.01036.x 11710762

[B66] Fritzsch G Boehme MU Thorndyke M Nakano H Israelsson O Stach T Schlegel M Hankeln T Stadler PF PCR survey of *Xenoturbella bocki* Hox genes J Exp Zool B Mol Dev Evol 2008 310B 278 284 10.1002/jez.b.21208 18161857

[B67] Rouse GW Jermiin LS Wilson NG Eeckhaut I Lanterbecq D Oji T Young CM Browning T Cisternas P Helgen LE Stuckey M Messing GC Fixed, free, and fixed: the fickle phylogeny of extant Crinoidea (Echinodermata) and their Permian-Triassic origin Mol Phylogenet Evol 2013 66 161 181 10.1016/j.ympev.2012.09.018 23063883

[B68] Burke AC Nowicki JL Hox genes and axial specification in vertebrates Am Zool 2001 41 687 697 10.1668/0003-1569(2001)041[0687:HGAASI]2.0.CO;2

[B69] Wellik DM Hox genes and vertebrate axial pattern Curr Top Dev Biol 2009 88 257 278 19651308 10.1016/S0070-2153(09)88009-5

[B70] Schilling TF Knight RD Origins of anteroposterior patterning and Hox gene regulation during chordate evolution Philos T Roy Soc B 2001 356 1599 1613 10.1098/rstb.2001.0918 PMC1088539 11604126

[B71] Wellik DM Capecchi MR *Hox10* and *Hox11* genes are required to globally pattern the mammalian skeleton Science 2003 301 363 367 10.1126/science.1085672 12869760

[B72] Alexander T Nolte C Krumlauf R Hox Genes and Segmentation of the hindbrain and axial skeleton Annu Rev Cell Dev Bi 2009 25 431 456 10.1146/annurev.cellbio.042308.113423 19575673

[B73] Mungpakdee S Seo HC Angotzi AR Dong XJ Akalin A Chourrout D Differential evolution of the 13 Atlantic salmon Hox clusters Mol Biol Evol 2008 25 1333 1343 10.1093/molbev/msn097 18424774

[B74] Wada H Satoh N Patterning the protochordate neural tube Curr Opin Neurobiol 2001 11 16 21 10.1016/S0959-4388(00)00168-9 11179867

[B75] Horigome N Myojin M Ueki T Hirano S Aizawa S Kuratani S Development of cephalic neural crest cells in embryos of *Lampetra japonica*, with special reference to the evolution of the jaw Dev Biol 1999 207 287 308 10.1006/dbio.1998.9175 10068464

[B76] Oulion S Borday-Birraux V Debiais-Thibaud M Mazan S Laurenti P Casane D Evolution of repeated structures along the body axis of jawed vertebrates, insights from the *Scyliorhinus canicula* Hox code Evol Dev 2011 13 247 259 10.1111/j.1525-142X.2011.00477.x 21535463

[B77] Davis A Stellwag EJ Spatio-temporal patterns of Hox paralog group 3-6 gene expression during Japanese medaka (*Oryzias latipes*) embryonic development Gene Expr Patterns 2010 10 244 250 10.1016/j.gep.2010.05.003 20471497

[B78] McIntyre DC Rakshit S Yallowitz AR Loken L Jeannotte L Capecchi MR Wellik DM Hox patterning of the vertebrate rib cage Development 2007 134 2981 2989 10.1242/dev.007567 17626057

[B79] Mallo M Wellik DM Deschamps J Hox genes and regional patterning of the vertebrate body plan Dev Biol 2010 344 7 15 10.1016/j.ydbio.2010.04.024 20435029 PMC2909379

[B80] Canestro C Bassham S Postlethwait J Development of the central nervous system in the larvacean *Oikopleura dioica* and the evolution of the chordate brain Dev Biol 2005 285 298 315 10.1016/j.ydbio.2005.06.039 16111672

[B81] Castro LFC Rasmussen SLK Holland PWH Holland ND Holland LZ A Gbx homeobox gene in amphioxus: insights into ancestry of the ANTP class and evolution of the midbrain/hindbrain boundary Dev Biol 2006 295 40 51 10.1016/j.ydbio.2006.03.003 16687133

[B82] Wada H Saiga H Satoh N Holland PWH Tripartite organization of the ancestral chordate brain and the antiquity of placodes: insights from ascidian Pax-2/5/8: Hox and Otx genes Developmen 1998 125 1113 1122 10.1242/dev.125.6.1113 9463358

[B83] Jiang D Smith WC An ascidian engrailed gene Dev Genes Evol 2002 212 399 402 10.1007/s00427-002-0255-y 12436970

[B84] Ikuta T Saiga H Dynamic change in the expression of developmental genes in the ascidian central nervous system: revisit to the tripartite model and the origin of the midbrain-hindbrain boundary region Dev Biol 2007 312 631 643 10.1016/j.ydbio.2007.10.005 17996862

[B85] Kozmik Z Holland ND Kalousova A Paces J Schubert M Holland LZ Characterization of an amphioxus paired box gene, AmphiPax2/5/8: developmental expression patterns in optic support cells, nephridium, thyroid-like structures and pharyngeal gill slits, but not in the midbrain-hindbrain boundary region Development 1999 126 1295 1304 10021347 10.1242/dev.126.6.1295

[B86] Jackman WR Kimmel CB Coincident iterated gene expression in the amphioxus neural tube Evol Dev 2002 4 366 374 10.1046/j.1525-142X.2002.02022.x 12356266

[B87] Schubert M Holland ND Laudet V Holland LZ A retinoic acid-Hox hierarchy controls both anterior/posterior patterning and neuronal specification in the developing central nervous system of the cephalochordate amphioxus Dev Biol 2006 296 190 202 10.1016/j.ydbio.2006.04.457 16750825

[B88] Wada H Garcia-Fernandez J Holland PWH Colinear and segmental expression of amphioxus Hox genes Dev Biol 1999 213 131 141 10.1006/dbio.1999.9369 10452851

[B89] Koop D Holland ND Semon M Alvarez S de Lera AR Laudet V Holland LZ Schubert M Retinoic acid signaling targets Hox genes during the amphioxus gastrula stage: insights into early anterior-posterior patterning of the chordate body plan Dev Biol 2010 338 98 106 10.1016/j.ydbio.2009.11.016 19914237

[B90] Amemiya CT Prohaska SJ Hill-Force A Cook A Wasserscheid J Ferrier DEK Pascual-Anaya J Garcia-Fernandez J Dewar K Stadler PF The amphioxus Hox cluster: Characterization, comparative genomics, and evolution J Exp Zool B Mol Dev Evol 2008 310B 465 477 10.1002/jez.b.21213 18351584

[B91] Lowe CJ Wu M Salic A Evans L Lander E Stange-Thomann N Gruber CE Gerhart J Kirschner M Anteroposterior patterning in hemichordates and the origins of the chordate nervous system Cell 2003 113 853 865 10.1016/S0092-8674(03)00469-0 12837244

[B92] Gerhart J Lowe C Kirschner M Hemichordates and the origin of chordates Curr Opin Genet Dev 2005 15 461 467 10.1016/j.gde.2005.06.004 15964754

[B93] Popodi E Kissinger JC Andrews ME Raff RA Sea urchin Hox genes: Insights into the ancestral Hox cluster Mol Biol Evol 1996 13 1078 1086 10.1093/oxfordjournals.molbev.a025670 8865662

[B94] Lowe CJ Wray GA Radical alterations in the roles of homeobox genes during echinoderm evolution Nature 1997 389 718 721 10.1038/39580 9338781

[B95] David B Mooi R Embryology supports a new theory of skeletal homologies for phylum Echinodermata Cr Acad Sci III-Vie 1996 319 577 584

[B96] Mooi R David B Radial symmetry, the anterior/posterior axis, and echinoderm Hox genes Annu Rev Ecol Evol S 2008 39 43 62 10.1146/annurev.ecolsys.39.110707.173521

[B97] Arenas-Mena C Martinez P Cameron RA Davidson EH Expression of the Hox gene complex in the indirect development of a sea urchin Proc Natl Acad Sci U S A 1998 95 13062 13067 10.1073/pnas.95.22.13062 9789041 PMC23710

[B98] Nielsen MG Popodi E Minsuk S Raff RA Evolutionary convergence in *Otx* expression in the pentameral adult rudiment in direct-developing sea urchins Dev Genes Evol 2003 213 73 82 12632176 10.1007/s00427-003-0299-7

[B99] Steinmetz PRH Kostyuchenko RP Fischer A Arendt D The segmental pattern of *otx*, *gbx*, and Hox genes in the annelid *Platynereis dumerilii* Evol Dev 2011 13 72 79 10.1111/j.1525-142X.2010.00457.x 21210944

[B100] Acampora D Gulisano M Simeone A Genetic and molecular roles of Otx homeodomain proteins in head development Gene 2000 246 23 35 10.1016/S0378-1119(00)00070-6 10767524

[B101] Buresi A Baratte S Da Silva C Bonnaud L: *orthodenticle/otx* ortholog expression in the anterior brain and eyes of *Sepia officinalis* (Mollusca, Cephalopoda) Gene Expr Patterns 2012 12 109 116 10.1016/j.gep.2012.02.001 22365924

[B102] Peterson KJ Arenas-Mena C Davidson EH The A/P axis in echinoderm ontogeny and evolution: evidence from fossils and molecules Evol Dev 2000 2 93 101 10.1046/j.1525-142x.2000.00042.x 11258395

[B103] Urbach R A procephalic territory in *Drosophila* exhibiting similarities and dissimilarities compared to the vertebrate midbrain/hindbrain boundary region Neural Dev 2007 2 23 10.1186/1749-8104-2-23 17983473 PMC2206033

[B104] Denes AS Jekely G Steinmetz PRH Raible F Snyman H Prud'homme B Ferrier DEK Balavoine G Arendt D Molecular architecture of annelid nerve cord supports common origin of nervous system centralization in Bilateria Cell 2007 129 277 288 10.1016/j.cell.2007.02.040 17448990

[B105] Schmerer M Savage RM Shankland M Pax beta: a novel family of lophotrochozoan Pax genes Evol Dev 2009 11 689 696 10.1111/j.1525-142X.2009.00376.x 19878290

[B106] Mooi R David B Wray GA Arrays in rays: terminal addition in echinoderms and its correlation with gene expression Evol Dev 2005 7 542 555 10.1111/j.1525-142X.2005.05058.x 16336408

[B107] Ferrier DEK Minguillon C Cebrian C Garcia-Fernandez J Amphioxus Evx genes: Implications for the evolution of the midbrain-hindbrain boundary and the chordate tailbud Dev Biol 2001 237 270 281 10.1006/dbio.2001.0375 11543613

[B108] Yu JK Satou Y Holland ND Shin-I T Kohara Y Satoh N Bronner-Fraser M Holland LZ Axial patterning in cephalochordates and the evolution of the organizer Nature 2007 445 613 617 10.1038/nature05472 17237766

[B109] Thaeron C Avaron F Casane D Borday V Thisse B Thisse C Boulekbache H Laurenti P Zebrafish *evx1* is dynamically expressed during embryogenesis in subsets of interneurones, posterior gut and urogenital system Mech Develop 2000 99 167 172 10.1016/S0925-4773(00)00473-1 11091087

[B110] Lemons D McGinnis W Genomic evolution of Hox gene clusters Science 2006 313 1918 1922 10.1126/science.1132040 17008523

[B111] Mooi R David B Skeletal homologies of echinoderms The Paleontological Society Papers 1997 3 305 335

[B112] David B Mooi R Contributions of the extraxial-axial theory to understanding the echinoderms B Soc Geol Fr 1999 170 91 101

[B113] David B Lefebvre B Mooi R Parsley R Are homalozoans echinoderms? An answer from the extraxial-axial theory Paleobiology 2000 26 529 555 10.1666/0094-8373(2000)026<0529:AHEAAF>2.0.CO;2

[B114] Ikuta T Saiga H Organization of hox genes in ascidians: present, past, and future Dev Dynam 2005 233 382 389 10.1002/dvdy.20374 15844201

